# Regulation of Notch1 Signalling by Long Non-Coding RNAs in Cancers and Other Health Disorders

**DOI:** 10.3390/ijms241612579

**Published:** 2023-08-08

**Authors:** Joanna Kałafut, Arkadiusz Czerwonka, Karolina Czapla, Alicja Przybyszewska-Podstawka, Justyna Magdalena Hermanowicz, Adolfo Rivero-Müller, Lidia Borkiewicz

**Affiliations:** 1 Department of Biochemistry and Molecular Biology, Medical University of Lublin, Aleje Raławickie 1, 20-059 Lublin, Poland; joannakalafut@umlub.pl (J.K.); arkadiuszczerwonka@umlub.pl (A.C.); karolinadudziak@umlub.pl (K.C.); alicja.przybyszewska-podstawka@umlub.pl (A.P.-P.); 2 Department of Pharmacodynamics, Medical University of Bialystok, Mickiewicza 2C, 15-222 Bialystok, Poland; justyna.hermanowicz@umb.edu.pl; 3 Department of Clinical Pharmacy, Medical University of Bialystok, Waszyngtona 15, 15-274 Bialystok, Poland

**Keywords:** lncRNA, microRNA, Notch signalling, NOTCH, cancer

## Abstract

Notch1 signalling plays a multifaceted role in tissue development and homeostasis. Currently, due to the pivotal role of Notch1 signalling, the relationship between NOTCH1 expression and the development of health disorders is being intensively studied. Nevertheless, Notch1 signalling is not only controlled at the transcriptional level but also by a variety of post-translational events. First is the ligand-dependent mechanical activation of NOTCH receptors and then the intracellular crosstalk with other signalling molecules—among those are long non-coding RNAs (lncRNAs). In this review, we provide a detailed overview of the specific role of lncRNAs in the modulation of Notch1 signalling, from expression to activity, and their connection with the development of health disorders, especially cancers.

## 1. Introduction

The Notch receptors belong to a highly conserved family of transmembrane receptors responsible for transmitting intracellular signals upon cell-to-cell (juxtacrine) contact. One of the most characteristic features of the Notch receptors is that they act as membrane-anchored mechanosensing receptors coupled with a specific nuclear transcriptional modulator—the Notch intracellular domain (NICD) [1]. Notch ligands (Delta-like type ( DLL) and Jagged/Serrate proteins (JAG)), presented by a signal-sending cell, activate the Notch receptors (NOTCH-1, -2, -3, and -4) on the signal-receiving cell [2]. The canonical Notch signalling pathway involves two neighbouring cells; the receptor–ligand interaction results in mechanical forces that unveil a receptor’s proteolytic site near the plasma membrane, recognised and cleaved by the ADAM family of metalloproteinases (Site-2 cleavage; S2), triggering a series of proteolytic events that lead to the release of the NICD, upon gamma-secretase (γ-secretase) cleavage (Site-3; S3) [3]. The NICD is subsequently translocated to the nucleus, where it binds CSL (also known as CBF-1/RBP-jκ, Su (H), or Lag-1) and forms a transcriptional complex that regulates the expression of downstream genes, such as *HES1* (*Hes family bHLH transcription factor 1*), *HEY1* (*Hes-related family bHLH transcription factor with YRPW motif 1*), *MYC* (MYC proto-oncogene, bHLH transcription factor)*,* and *NRARP* (*NOTCH-regulated ankyrin repeat protein*) [4]. The final gene expression patterns and cell fate after Notch activation are highly varied and dependent on various factors such as the cell type, environment, pattern of activation, concentration of receptors and ligands on cell surfaces, and even the duration and strength of activation [5,6]. Such a plasticity in Notch signalling transmission is largely the result of modulation at multiple levels.

The earliest discovery of NOTCH physiological function was the characteristic “notches” found in the distal tip of *Drosophila melanogaster* wings in *Notch*-null mutants, among others, by Thomas Hunt Morgan, Calvin B. Bridges, and John S. Dexter at the beginning of the 20th century [7,8]. Since then, and thanks to advances in genetics and molecular biology, the Notch signalling system has been rapidly unravelled, mainly in *D. melanogaster* and *Caenorhabditis elegans* [9]. It is now clear that Notch signalling plays a key role in developmental and physiological functions. Some of the best described roles of NOTCH1 are the maintenance of stem cell populations [10] and the hippocampal [11] and olfactory [12] plasticity of the nervous systems. Yet it also plays defined functions in angiogenesis [13,14,15,16,17,18] and osteogenesis [19,20], among multiple other developmental processes. The Notch system is important in the regulation of embryonic development, such as during lateral inhibition and induction, binary cell fate, and boundary formation [21,22]. In fact, the Notch pathway is already active in mice embryos that consist only of four cells [23], and it is present in a variety of tissues and organs, such as the thymus [24], vascular system [25], and bone tissue [26], throughout the entire life span. The regulation of Notch activity is performed by transcriptional enhancers, silencers, and enzymes introducing posttranslational modifications, as well as partnering molecules. Aberrations of the multifaceted Notch system result in multiple health disorders, including cancers.

In humans, activating mutations of NOTCH1 was first associated with the development and progression of leukaemia (see also 2.1) [27,28,29,30]. High NOTCH1 activity has been also linked with several other types of cancers including lymphomas [27,28,29,30] and brain [31], breast [32], lung [33,34], ovarian [35], renal [36], and hepatocellular cancer [37,38]. The relationship between the Notch signalling pathway and cancer progression is complex and not only related to an acceleration of cell proliferation. NOTCH1 target genes are also associated with the control of apoptosis, epithelial–mesenchymal transition (EMT), increased drug resistance [39,40,41,42,43,44], modulation of the tumour microenvironment, and maintenance of the cancer stem cell population [45,46]. For these reasons, excessive activation or inhibition of Notch signalling can lead to a serious dysregulation of homeostasis. NOTCH1 activity has both oncogenic as well as tumour-suppressive effects on cells during oncogenesis, depending on the tumour type (for review, see [47]). The oncogenic role of NOTCH1 signalling is also connected with the dysregulation of cellular metabolism and genome stability [48,49].

The dynamic fine-tuning of NOTCH signalling is achieved by post-transcriptional and post-translational regulation. The first includes the control of RNA stability by RNA-binding proteins and microRNAs [50,51]. For example, *NOTCH1* mRNA stability is modulated by the methylation of adenine (m^6^A) residues by N^6^-methyladenosine methyltransferases such as METTL3 and METTL14 [52,53]. These modifications are recognized by proteins such as YTHDF2 (YTH *domain family 2*) that cause the inhibition of Notch signalling by the downregulation of *NOTCH1*, *HES1*, and *HES5* mRNA levels [50].

On the other hand, NOTCH1 is regulated via post-translational modifications (PTMs), meaning an addition of different functional groups, to the target amino acid side chain, which reversibly modulate the structure, activity, localisation, and stability of the target. NOTCH1 is regulated by several PTMs including proteolysis, phosphorylation, acetylation, methylation, hydroxylation, sumoylation, ubiquitination, and *O*-glycosylation [54]. For example, tumour-suppressor cyclin C-dependent kinases, CDK3, 8, and 19, phosphorylate the N1ICD at multiple sites, triggering the binding of Fbw7 ubiquitin ligase and further polyubiquitination and proteolytic degradation [55]. Many kinases also directly control N1ICD transcriptional activity, one of which is casein kinase 2 (CK2). First, it targets S1900 and, further, T1897 of the N1ICD, leading to a decrease in binding between the N1ICD and MAML (Mastermind)–CSL complex, therefore lowering transcriptional activity [56]. Additionally, the phosphorylation of the N1ICD at the second NLS is important for both nuclear localisation [57,58] and transcriptional activity [57], while murine double minute 2 (MDM2)-mediated ubiquitination was shown to enhance N1ICD transcriptional activity [59]. 

The regulatory mechanisms of Notch signalling also include the activity of ncRNAs, which encompass constitutively expressed ribosomal (rRNAs), transfer (tRNAs), small nuclear (snRNAs), and small nucleolar (snoRNA) RNAs, telomerase RNA (TERC), tRNA-derived fragments (tRFs), tRNA halves (tiRNAs), and also regulatory ncRNAs including microRNAs (miRNAs), Piwi-interacting RNAs (piRNAs), small interfering RNAs (siRNAs), circular RNAs (circRNAs), enhancer RNAs (eRNAs), and long non-coding RNAs (lncRNAs) [60]. The interaction between miRNAs and the Notch pathway has been widely reported and reviewed (for example, [61,62,63]); therefore, here, we will focus on lncRNAs and summarise how they affect transcriptional, translational, and post-translational Notch1 signalling.

## 2. Non-Coding RNAs as a Pivotal Control Factor of Notch Signalling

Numerous studies have documented the significant contribution of ncRNAs in the regulation of NOTCH1-related genes, including tumour suppressors and oncogenes. NcRNAs are roughly classified based on the length of the nucleotide sequences, ncRNA position relative to the target gene, and/or ncRNA function. The small ncRNAs (sncRNAs), of less than 200 nucleotides in length, include mainly miRNAs, siRNAs, piRNAs, tRFs, and tiRNAs, while lncRNAs are transcripts with lengths exceeding 200 nucleotides that are usually synthesised by the antisense transcription of target genes (antisense lncRNA) or within intergenic (lincRNA) or intronic loci [64,65]. LncRNAs assist in the remodelling of the genome architecture, gene transcription, and post-transcriptional RNA processing via a direct interaction with other nucleic acids and proteins [66]. As a result, they are involved in processes such as the control of the transcription [67] and translation [68], as well as the formation of nuclear subcomponents by DNA looping [69] and chromatin organisation [70]. Mechanistically, lncRNAs act as scaffolds or decoys for other RNAs and proteins to bind to. The ability of lncRNA to specifically interact with other nucleic acids is harnessed for the formation of transcriptional and chromatin modification complexes, and they also act as sponges for other ncRNAs. Subsequently, lncRNAs supervise other ncRNAs by controlling miRNA availability, mRNA maturation, and siRNA formation. All of these activities allow lncRNAs to oversee gene expression by controlling the function, mutual interactions, and intracellular localisation of proteins and other RNAs [71,72,73].

### 2.1. Controlling Transcription of NOTCH1 and NOTCH1-Related Genes in Cancers

The evolutionarily conserved elements of the Notch pathway are presented throughout the entire Animalia kingdom. Phylogenetic studies suggest that the fundamental molecules of the pathway, the NOTCH receptors and ligands, have played essential roles in animal evolution [74]. In humans, four independent *NOTCH* (1, 2, 3, 4) genes located on the 9q34.3, 1p12, 19p13.12, and 6p21.32 chromosomal regions, respectively, encode NOTCH1-4 proteins which are relatively similar in their core structure. The Notch ligands are encoded by five genes: *DLL* (1, 3, 4) and *JAG* (1, 2) [75]. The expression patterns of NOTCH receptor genes and their ligands depend on the specific cellular context and are often altered in a variety of tumours [48,76]. Moreover, in feedback regulation, activated NOTCH1 affects its own expression as well as expression of other NOTCH receptors and their ligands [77].

**Figure 1:**
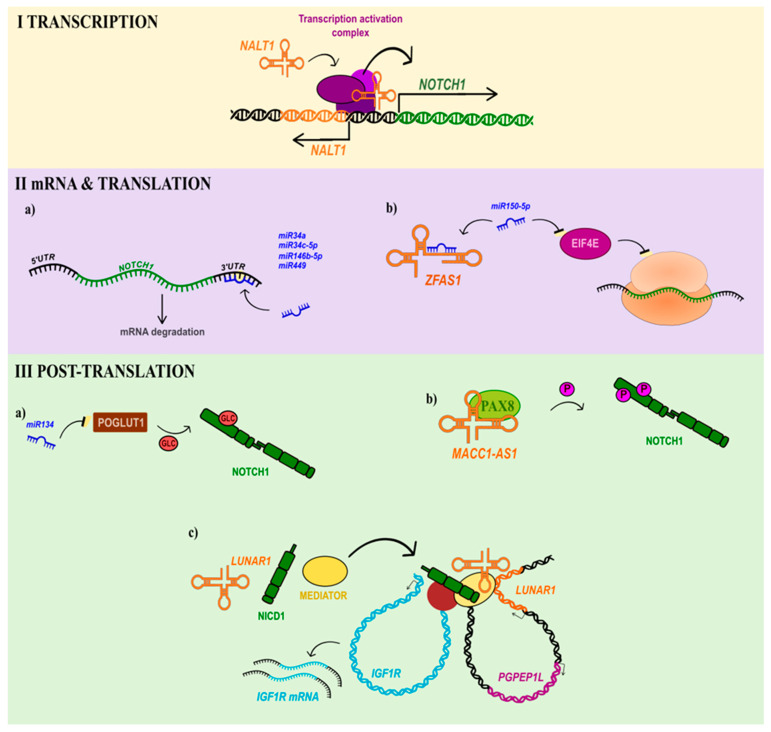
Models of ncRNA action in the control of the Notch1 pathway at the (**I**) transcriptional, (**II**) mRNA and translational, and (**III**) post-translational levels. (**I**) LncRNAs work with various RNA binding proteins (RBPs) to act as a transactivation element for *cis*-regulatory sequences (such as promoters, enhancers, suppressors), e.g., *NALT1* recruits to the *NOTCH1*-promoter transcriptional activation complex promoting Notch signalling in T-ALL. (**IIa**) Several miRNAs have been shown to bind the 3′-UTR of *NOTCH1* mRNA leading to its degradation. (**IIb**) LncRNAs such as *ZFAS1* sequestrate miRNAs, acting as miRNA sponges, i.e., the binding of *miR150-5p* prevents it from inactivating *eIF4E* mRNA and inhibition of the translation of NOTCH1 via blocking the translation initiation step. (**III**) NcRNAs affect the activity and stability of NOTCH1 by regulating the expression of proteins that can modify NOTCH1 protein, such as (**IIIa**) POGLUT1, responsible for NOTCH1 glycosylation, or (**IIIb**) PAX8, controlling the NOTCH1 phosphorylation status. (**IIIc**) LncRNA *LUNAR1* reorganises chromatin in close proximity to its own locus and binds the NOTCH1 and Mediator complex to enhance *IGF1R* transcription.

Various lncRNAs act as inhibitors or activators of Notch signalling elements. For example, *NOTCH1* expression is regulated, at the transcriptional level, by neighbouring genes, such as lncRNA *RP11-611D20.2* (also known as *LINC01573*) which acts as a *cis* transcriptional activator of NOTCH1 signalling in paediatric T cell acute lymphoblastic leukaemia (T-ALL) and is therefore named *NALT1* (*NOTCH1-associated lncRNA in T-ALL*) (Figure 1(I)) [78]. Recently, a similar role of *NALT1* was reported in gastric cancer (GC), where the knockdown of this lncRNA resulted in a decrease in *NOTCH1* expression, which reduced the invasion and migration of GC cells [79].

The effect of lncRNAs on the chromatin organisation affecting the Notch1 pathway has also begun to be elucidated in breast cancer (MDA-MB-231) cells. The silencing of the highly expressed intergenic lncRNA regulator of reprogramming (*linc-ROR)* led to an increase in NOTCH1, LC3-II (LC3-phosphatidylethanolamine conjugate), Beclin-1, and p53 expression, promoting autophagy and apoptosis [40]. Mechanistically, *linc-ROR* decreases the expression of *miR-34a*, a *NOTCH1* mRNA inhibitor, via the inhibition of histone H3 acetylation in the *miR-34a* promoter region. Similar epigenetic phenomena were described in human cholangiocarcinoma (CCA), in which Enhancer of zeste homolog 2 (EZH2)-mediated histone 3 trimethylation of lysine 27 (H3K27me3) in the same promoter lowered *miR-34a* levels and promoted Notch1 signalling [80].

In the control of gene expression, lncRNAs act also via the regulation of proteins affecting *NOTCH1*. Studies on obesity have shown that NK6 homeobox 1 (Nkx6.1), which binds *NOTCH1* at a 139 bp enhancer sequence (known as the CR2 fragment) in the second intron and positively regulates its expression [81], is upregulated by lncRNA *regulator of insulin transcription ROIT* [82]. *ROIT* interacts with and induces the ubiquitination and degradation of DNA methyltransferase 3a via the proteasome, thereby reducing methylation of the *Nkx6.1* (*NK6 homeobox 1*) promoter and consequently increasing the expression of Nkx6.1 and insulin genes [82]. These data support the importance of the *ROIT*/Nkx6.1/Notch1 pathway in diabetes.

### 2.2. NOTCH1 mRNA and Translation Control

Increasing evidence indicates that both lncRNAs and miRNAs regulate *NOTCH1* mRNA processing. Most of these interactions have been described in stem cells and various diseases including cancer, where aberrant ncRNA levels result in the dysregulation of NOTCH1 signalling.

*NOTCH1* mRNA is tightly controlled by miRNAs binding to its 3′ UTR region; however, lncRNAs can efficiently prevent such interactions as described for *FEZF1-AS1* in non-small-cell lung cancer (NSCLC) [83] and *SNHG7* in breast cancer [84], acting as *miR-34a* sponges sequestering this miRNA from its target mRNAs, consequently increasing the amount of *NOTCH1* mRNA. A similar sponge activity was reported for *NEAT1* (*nuclear-enriched abundant transcript 1*) against *miR-146b-5p* in T-ALL [85], *LncND* (*neurodevelopment*) and *miR-143-3p* in neuronal development [86], *LINC01123* and *miR-449b-5p* in renal cell carcinoma [87], an intergenic lncRNA *346* (*LINC00346*) and *miR-34a-5p* in GC [88], *HCG18* and *miR-34c-5p* in bladder cancer [89], and *DCST1-AS1* binding *miR-92a-3p* in endometrial carcinoma (EC) [90]. Contrarily, lncRNA *CARMEN7* (*cardiac mesoderm enhancer-associated non-coding RNA*), which is increased downstream of NOTCH activation [91], augments *miR-143/145* expression through an enhancer element located in this microRNA’s locus, thereby promoting the differentiation of adult human cardiac precursor cells into smooth muscle cells [92].

A slightly different mode of *NOTCH1* regulation via lncRNAs has been reported in head and neck squamous cell carcinoma (HNSCC) patients, whose low expression of lncRNA *ZFAS1* (*ZNFX1 antisense RNA 1*) correlated with the upregulation of *NOTCH1* and better survival, suggesting an oncogenic role of this lncRNA. Yet the *ZFAS1* levels typically differed depending on the cancer stage and tumour size (T-stage). It was shown that *ZFAS1* overexpression in HNSCC cells and tissues samples is associated with poor patient outcomes [93]. At the molecular level, *ZFAS1* binds *miR-150-5p*, the inhibitor of eIF4E (eukaryotic translation initiation factor 4E), which is required for the translation of several genes involved in proliferation, survival, EMT, and cancer invasion [93]. A year later, studies on the inflammatory response and apoptosis of RAW264.7 macrophages revealed that *miR-150-5p* alleviates those processes, at least partially, via Notch1 targeting. The results highlighted *miR-150-5p* as a target in the development of anti-inflammatory and anti-apoptotic drugs for sepsis treatment [94].

Zhao and collaborators have shown that the presence of *DLX6 antisense RNA 1* (*DLX6-AS1*) is associated with the Notch1 signalling pathway. The knockdown of this lncRNA led to a reduction in Notch1 signalling by diminishing *NOTCH1*, *p21*, and *HES1* at the mRNA and protein levels. Clinical analyses indicated that a high level of *DLX6-AS1* in patients with epithelial ovarian cancer was significantly associated with lymph node metastasis and a poor prognosis [95]. Furthermore, the role of *DLX6-AS1* has been reported as tumour-promoting in pancreatic cancer [96], non-small-cell lung cancer [97], and glioma [98] through inhibiting *miR-181b*, *miR-144*, and *miR-197-5p*, respectively, yet their association with the Notch1 signalling pathway has not been investigated in detail.

In addition to regulation by lncRNAs and miRNAs, the *NOTCH1* transcript can also be backspliced into a circular RNA (circ-*NOTCH1*) during splicing, which has both sponge and decoy activities. For example, circ-*NOTCH1* binds METTL14, depletes the amount of free METTL14, and consequently maintains a high level of *NOTCH1* mRNA [52].

### 2.3. Controlling Notch1 Signalling by lncRNA at the Post-Translational Level in Cancers

LncRNAs often function as scaffolds for signalling proteins, and the Notch pathway is no exception. Several lncRNAs control Notch1 signalling both by directly interacting with the NOTCH1 protein, such as lncRNA *LINC00511* which mediates contacts between the N1ICD transcriptional complex and enhancers, e.g., at the *SOX9* gene [99], or by controlling other proteins that are involved in NOTCH1 activity and processing.

*Neighbour of BRCA1 gene* 2 (*NBR2*) is a lncRNA that directly binds to the N1ICD, as shown in osteosarcoma (OS) [100]. The mechanism of action for *NBR2* is not yet well established; however, it seems to play an essential role in the development of Notch-dependent OS. OS patients with low *NBR2* expression have a shorter overall survival rate compared with patients with higher *NBR2* levels. Additionally, the overexpression of this lncRNA decreased *NOTCH1*, N-cadherin (*CDH2*), and *Vimentin* expression and led to the inhibition of cell migration and proliferation but did not affect apoptosis, both in OS [100] and in NSCLC [101].

As previously mentioned, lncRNAs appear to be strongly associated with the development of T-ALL. The first reports linking human NOTCH1 and carcinogenesis were described in the early 1990s and were related to NOTCH1 chromosomal translocations in T-ALL [102]. Several years later, the genome-wide mapping of NOTCH1-regulated transcripts in T-ALL revealed 182 lncRNAs, among which 55% were interacting with the N1ICD–RBPJΚ (*recombination signal binding protein for immunoglobulin Kappa J region*, also known as CSL) activator complex. One of these lncRNAs is *LUNAR1* (*leukaemia-induced non-coding activator RNA 1*), acting as an enhancer of the expression of its neighbouring gene, *IGF1R* (*insulin-like growth factor1-receptor*), which is essential for T-ALL tumour development in vitro and in vivo [103]. As *LUNAR1* binds to enhancer elements in the promoter of *IGF1R*, its own promoter, and the N1ICD, it was suggested that it exploits the chromatin configuration to recruit the mediator complex and sustain the full activation of the *IGF1R* promoter, fuelling T-ALL development (Figure 1(IIIc)) [103]. In agreement with this, it has been found that the inhibition of *LUNAR1* in CRC decreases tumour growth and efficiently depletes IGF1 pathway activity, indicating a role for the *LUNAR1*/NOTCH1/IGF1R axis in cancer development [104].

**Figure 2:**
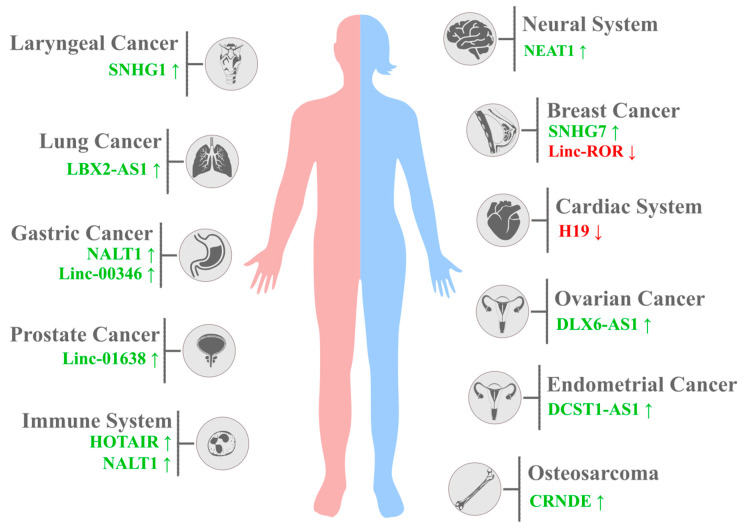
Cancer-related lncRNA overexpression and its impact on the regulation of NOTCH1 expression. LncRNAs upregulating NOTCH1 expression are marked in green and with ↑; lncRNAs downregulating NOTCH1 expression are marked in red and with ↓.

LncRNAs may also modulate Notch1 signalling indirectly by binding proteins related to NOTCH1, such as the Paired box gene 8 (PAX8) protein which was proposed to activate the N1ICD in the nucleus by modulating its phosphorylation status and affecting the transcription of its target genes, as well as promoting aerobic glycolysis in pancreatic carcinoma. PAX8 directly interacts with lncRNA *MACC1-AS1* (*MACC1 antisense RNA 1*), increasing its stability, and therefore modulates its activity. The knockdown of *MACC1-AS1* inhibited cancer proliferation and metastasis [105]. Based on these facts, *MACC-AS1*/PAX8/Notch1 signalling might be considered as a target for the alternative treatment of pancreatic carcinoma patients. The recently described functional role of lncRNA interaction with the NOTCH1 receptor is summarised in Figure 2 and Supplementary Table S1.

## 3. Notch1-Related lncRNAs and Other Diseases

As the Notch system controls multiple processes, it appears to be tightly related to some congenital and developmental health disorders. Besides cancers, among the dysfunctions linked with abnormal Notch1 signalling are muscle, bone, cerebrovascular, and neurodegenerative diseases. This includes vascular diseases like strokes and ischemic attacks [106], thoracic aortic aneurysms [107], heart malfunction [108], neurodegenerative diseases including Alzheimer’s disease, multiple sclerosis and amyotrophic lateral sclerosis [109], pulmonary dysfunctions [110], severe vertebral abnormalities in spondylocostal dysostosis and spondylothoracic dysostosis [111], disorders related to the dysfunction of muscle, like Duchenne muscular dystrophy [112], bone structure dysfunction, like Klippel–Feil syndrome [113], as well as Hajdu–Cheney [114], Adams–Oliver [115], and Von Hippel–Lindau [116], and multiorgan dysfunction like Alagille syndrome [117]. Still, in many cases, the relation between NOTCH1 signalling and the development or risk of various diseases remains unsettled. Nevertheless, there is mounting evidence linking abnormalities in the lncRNA-mediated control of *NOTCH1* to various levels of its expression in several health disorders (see Table 1).

Among the lncRNAs involved in the epigenetic regulation of Notch1 signalling in diseases are *HOTAIR* and *Potassium voltage-gated channel subfamily Qmember 1 overlapping transcript 1* (*KCNQ1OT1*) [118,119,120]. In autoimmune diseases, the overexpression of *HOTAIR* in both systemic sclerosis (SSc) myofibroblasts and SSc skin biopsies correlates with a reduction in *miRNA-34a* expression and consequent Notch pathway activation. Mechanistically, *HOTAIR* controls the EZH2 methyltransferase-dependent trimethylation of 27 lysine residues in histone H3 (H3K27me3) and therefore epigenetically represses *miRNA-34a* expression [118,121,122]. NOTCH1 is also associated with the development of myocardial infarction (MI). 

**Table 1:** Interaction between lncRNA and Notch1 signalling in health disorders.

Localisation	Disease	lncRNA	NOTCH1 Expression	Cell Line	Animal Models	Patients	Reference
Cardiac system	Acute myocardial infarction	XIST↑	↑	-	AMI rat model	-	[123]
	Calcific aortic valve disease	H19↑	↓	VICs, Saos2, COS7	-	36 patients	[124]
	Myocardial infarction	KCNQ1OT1↑	↑	-	C57BL/6 male mice	-	[120]
	Ischemic stroke	H19↑	↓	-	Male C57BL/6 J mice	40 patients	[125]
Immune system	T-ALL	NALT1↑	↑	Jurkat cells	-	Bone marrow of 20 children	[78]
	Systemic sclerosis (SSc)	HOTAIR↑	↑	-	-	12 adult patients	[118]
Neural system	Epilepsy	NEAT1↑	↑	CTX-TNA	-	6 patients	[126]
	Intervertebral disc degeneration	FAM83H-AS1↑	↑	-	-	10 patients	[127]
	Nasopharyngeal carcinoma	SNHG12↑	↑	SUNE1, CNE1, CNE2 68, HNE-1	-	139 tissue samples	[128]
Head and neck cancer	Oesophageal cancer	MALAT1↑	↑	TE-1, EC109, KYSE30, OE21	-	-	[129]
		SNHG1↑	↑	Eca109, TE-1	-	72 patients	[130]
	Laryngeal cancer	SNHG1↑	↑	-	-	42 patients (different tumour stages)	[131]
Digestive system cancer	Pancreatic carcinoma	MACC1-AS1↑	↑	BxPC-3, PANC-1, MIA PaCa-2, KP-2, AsPC-1, Capan-1	-	2 cohorts (98 and 124 patients) of primary tissues	[105]
	Gastric cancer	NALT1 (LINC01573)↑	↑	SGC-7901, BGC-823	-	336 patients after D2 lymph node dissected gastrectomy	[79]
		LINC00346↑ (sponge for miR-34a-5p)	↑	MGC803, BGC823, MKN28, MKN45, SGC7901	Xenografts in athymic (nu/nu) mice	58 gastric adenocarcinoma tissue samples	[88]
	Colorectal carcinoma	FAM83H-AS1↑	↑	SW480, LoVo, HCT116, HT29	-	40 patients	[132]
		MALAT1↑	↑	COLO205, HCT-116, LoVo, HT26, SW480	Nude Balb/c mice	-	[133]
	Hepatocellular carcinoma	LINC00261↓	↑	SMCC-7721, MHCC97L, MHCC97H	-	66 tissue samples	[134]
Reproductive tract	Ovarian cancer	DLX6-AS1↑	↑	HEY, SKOV3, OVCAR-3	-	128 tissue samples	[95]
		MALAT1↑	↑	A2780, OVCAR3, COC1, A2780/ CDDP, COC1/CDDP, OVCAR3/DDP	-	20 paired tumour tissue samples	[135]
	Endometrial carcinoma (EC)	MEG3↓	↑	HEC-1A, KLE	-	30 tumour tissue samples	[136]
		DCST1-AS1↑	↑	HEC-1	-	62 patients	[90]
	Prostate carcinoma	LINCO1638↑	↑	-	-	42 patients	[137]
		GHET1↑	↑	LNCap, C4-2	-	30 patients	[138]
Other cancers	Breast cancer	linc-ROR↑	↓	MDA-MB-231	-	-	[40]
		SNHG7↑	↑	-	-	37 pairs of tumour tissue samples	[84]
	Lung cancer	NBR2↓	↑	-	-	50 NSCLC patient tissue samples	[101]
		LBX2-AS1↑	↑	A549, PC9, H1975, SPC-A1, H1299	-	165 NSCLC patients	[139]
		EGFR-AS1↓	↑	NCH-H460, NCH-H23	-	87 NSCLC patients	[140]
		LET↓	↑	A549, 95D, NCI-H292, NCI-H1975	-	66 NSCLC patients	[141]
	Osteosarcoma	MEG3↓	↑	MG-63, U2OS	-	-	[142]
		NBR2↓	↑	MG-63, U2OS, SAOS-2	-	62 patients	[143]
		CRNDE↑	↑	MG-63, SAOS-2, U2OS	-	72 patients	[144]

Abbreviations: ↑ represents upregulation; ↓ represents downregulation.

In a mouse MI model, *KCNQ1OT1* was reported to affect Notch1 signalling via decreasing *RUNX3* (inhibitor of Notch1 signalling) expression through the methylation of its promoter [120]. Consequently, the overexpression of *KCNQ1OT1* led to the activation of the Notch1 signalling pathway and the development of MI. In other studies, Zhang et al., reported the regulation of NOTCH expression by X-inactive specific transcript (*XIST*) in acute MI in a rat model. *XIST* silencing led to a lower NOTCH1 expression level via increasing *miR-449*, which manifested in an inhibition of myocardial cell apoptosis and a reduction in the pathological injuries [123]. Thus, targeting *XIST*-NOTCH1 signalling might be considered as a potential therapeutic strategy for MI; however, more detailed research is needed as *XIST* silences the entire X chromosome and the described effect on *NOTCH1* might not be specific.

One of the first discovered eukaryotic lncRNAs, *H19*, was shown to silence *NOTCH1* transcription by preventing the recruitment of p53 to its promoter in calcific aortic valve disease (CAVD). Hadji et al., reported that increased levels of *H19* in CAVD lead to an abnormal mineralisation of the aortic valve [124]. Reducing NOTCH1 signalling in valve interstitial cells increases the expression of genes such as *RUNX2* and *BMP2* which play a pro-osteogenic role [124,145]. A similar mechanism of action of *H19* has been documented in studies of neurogenesis after ischemic stroke. In this case, the silencing of the *H19* lncRNA leads to an increase in *NOTCH1* transcription, which promotes the process of neurogenesis. Moreover, patients with elevated levels of circulating *H19* have a worse prognosis after ischemic stroke [125].

There is also evidence that lncRNAs regulate Notch1 signalling during viral infection. The host NOTCH1 downregulates the prototype foamy virus (PFV) internal promoter activity, which triggers the expression of the viral transcriptional transactivator (Tas), a protein essential for viral replication and gene expression. However, lncRNA *RP5* acts in opposition to NOTCH1 by promoting the expression of *miR-129-5p*, which knocks down *NOTCH1* mRNA, and, therefore, restoring Tas expression. This work provides evidence that some host lncRNAs promote PFV replication by outweighing NOTCH1 inhibition during early viral infection [146].

Data on the regulation of *NOTCH1* mRNA by ncRNAs in health disorders are in abundance. In studies on the development of epilepsy, the silencing of lncRNA *NEAT1* results in the downregulation of *NOTCH1*, *JAG1*, and *HES1*. *NEAT1* affects NOTCH1 signalling by suppressing *miR-129-5p*, which, as described above, targets *NOTCH1* mRNA [126].

## 4. Medicinal Perspectives for lncRNAs in Notch1-Related Diseases

A better understanding of the role of lncRNA on Notch1 signalling might provide new therapeutic strategies. The interconnection between Notch signalling and lncRNAs makes the latter potential biomarkers for Notch signalling activity, as they have high tissue- and tumour-type-specific expression patterns [147,148]. A clear advantage of lncRNAs as biomarkers is their fast and sensitive quantitative analysis by real-time RT-PCR, which can be performed from biological fluids [149,150,151]. The applicability of lncRNAs as biomarkers is well illustrated by *PCA3* (*prostate cancer gene 3*), routinely used in clinical applications as a prostate cancer marker [152], for instance, for patients who obtain a negative result from a prostate biopsy together with a raised level of prostate-specific antigen (PSA) which may indicate undiagnosed cancer [153]. To date, several lncRNAs have been shown to be associated with cancer progression and can be considered as survival factors for certain neoplasias. For example, the upregulation of lncRNAs *DCST1-AS1*, *NALT1*, *LBX2-AS1*, and *MACC1-AS1* correlates with the poor survival of patients with EC, GC, NSCLC, and pancreatic carcinoma, respectively [79,90,105,139]. Meanwhile, the levels of *SNHG1*, *NALT1*, and *CRNDE* are associated with metastases in EC, GC, and OS, respectively [79,130,144]. In colorectal cancer, the expression of lncRNA *CCAT1* (*colon cancer–associated transcript 1*) may potentially predict the response to JQ1, a chemical inhibitor of bromodomain containing four (BRD4) protein important for colorectal cancer proliferation [154]. At the molecular level, BRD4 recognises and interacts with acetylated histone tails, leading to chromatin remodelling by recruiting and stabilising multiprotein complexes to DNA [155,156]. As BRD4 plays an important role in mediating the expression of genes involved in cancers and non-cancer diseases, several drugs targeting it are currently in clinical trials [157].

Among those related to Notch1 signalling, a high *NALT1* expression level has also been detected in T-ALL patient samples [78]. The increased expression of *LINCO1638* lncRNA and *NOTCH1* has been observed in prostate carcinoma patients [137]. Cai et al., showed that NOTCH1-interacting lncRNA *NBR2* expression was downregulated in OS tissues and correlated with a shorter overall survival time compared with patients with higher *NBR2* expression [143]. Due to the specificity of lncRNAs, not only in cancer types but also within subtypes [148], they can serve as biomarkers for patient stratification as well as for drug response prediction. In gastric cancer cell lines (SGC7901/DDP and BGC823/DDP), NOTCH1 was shown to promote the evolution of cisplatin-resistant cells via the upregulation of lncRNA *AK022798* [158], while its downregulation using siRNAs reduced the expression of drug resistance genes. However, subsequent research is required to establish whether lncRNA may be better in predicting the responses of NOTCH inhibitors in relation to what has been discovered so far.

Other important modulators of *NOTCH1* are circular RNAs (circRNAs). Recent studies have shown the potential of circRNAs as prognostic biomarkers, for instance, *hsa_circ_0072309* [159] and *circCNOT2* [160] in breast cancer. Moreover, it has been shown that *NOTCH1* and the Notch1 signalling pathway could be upregulated via *circNFIX*, resulting in glioma progression [161]. *CircNFIX* acts by sponging *miR-34a-5p*, which targets *NOTCH1*. *Circ-ASH2L* also comes in handy in the diagnosis and progression of pancreatic ductal adenocarcinoma, as high *circ-ASH2L* expression was correlated with lymphatic invasion and the TNM (tumour, node, metastasis) stage, plus it was an independent risk factor for pancreatic patient survival. Similar to *circNFIX*, *circ-ASH2L* functions as a miRNA sponge for *miR-34a* and promotes tumour progression in vivo [162].

The circRNA research is still in its infancy, yet with the development of research strategies, effective clinical applications of circRNA will arise and expand in the diagnosis, treatment, and prognosis of cancer.

## 5. Conclusions and Future Perspectives

The efforts to understand the roles of Notch signalling in cancer have revealed various outcomes, either oncogenic or tumour suppressive depending on the cancer type and stage of tumourigenesis. This appears to be largely the result of complex crosstalks between numerous other signalling molecules and pathways. Here, we have reviewed the recent data on the role of lncRNAs in the tweaking of NOTCH1 activity via the regulation of the *NOTCH1* gene, mRNA, and protein. In head and neck tumours, such as HNSCC, where the absence of or reduction in NOTCH1 signalling is beneficial for tumour progression, the low expression of lncRNA *ZFAS1* (necessary for regulation of the translation of several cancer genes) resulted in the upregulation of *NOTCH1* and better survival [93], while in BC, where high NOTCH1 activity fuels tumour progression, *SNHG7* lncRNA promotes the expression of NOTCH1 and EMT by binding *miR-34a*, which causes malignant behaviour and increases the survival and proliferation of BC cells [84]. Therefore, the simple extrapolation of data from studies conducted on other cell types might be misleading in the case of Notch signalling. Also, more research discovering the network between miRNA, lncRNA, and circRNA is needed to further reveal the possible compensatory effects that may affect therapy.

In the case of Notch signalling, it is also important to note that these molecules might act differently on each NOTCH receptor, as each of them gives partially compensatory, partially unique, and partially opposite downstream responses. Thus, lncRNAs seem to be more selective, e.g., for targeting, than other molecular targets such as γ-secretase, which affects all NOTCH receptors and other signalling pathways [163].

Taken together, it is still too early to hypothesise whether lncRNA will be commonly used for therapies or diagnoses. The more we know about the functions of lncRNA, miRNA, and circRNA and their influences on a disease, the closer we are to their routine use in clinics.

## Data Availability

Not applicable.

## Supplementary Materials

The following supporting information can be downloaded at https://www.mdpi.com/article/10.3390/ijms241612579/s1.

## References

[1] Bray S.J. (2006). Notch signalling: A simple pathway becomes complex. Nat. Rev. Mol. Cell Biol..

[2] Cordle J., Johnson S., Tay J.Z.Y., Roversi P., Wilkin M.B., de Madrid B.H., Shimizu H., Jensen S., Whiteman P., Jin B. (2008). A conserved face of the Jagged/Serrate DSL domain is involved in Notch trans-activation and cis-inhibition. Nat. Struct. Mol. Biol..

[3] Kopan R., Ilagan M.X.G. (2009). The canonical Notch signaling pathway: Unfolding the activation mechanism. Cell.

[4] Henrique D., Schweisguth F. (2019). Mechanisms of Notch signaling: A simple logic deployed in time and space. Development.

[5] Nandagopal N., Santat L.A., LeBon L., Sprinzak D., Bronner M.E., Elowitz M.B. (2018). Dynamic Ligand Discrimination in the Notch Signaling Pathway. Cell.

[6] Shaya O., Binshtok U., Hersch M., Rivkin D., Weinreb S., Amir-Zilberstein L., Khamaisi B., Oppenheim O., Desai R.A., Goodyear R.J. (2017). Cell-Cell Contact Area Affects Notch Signaling and Notch-Dependent Patterning. Dev. Cell.

[7] Dexter J.S. (1914). The Analysis of a Case of Continuous Variation in Drosophila by a Study of Its Linkage Relations. The American Naturalist.

[8] Morgan T.H., Bridges C.B. (1919). The Inheritance of a Fluctuating Character. J. Gen. Physiol..

[9] Yamamoto S., Schulze K.L., Bellen H.J. (2014). Introduction to Notch Signaling. Methods in Molecular Biology (Clifton, N.J.).

[10] Bigas A., Espinosa L. (2016). Notch Signaling in Cell–Cell Communication Pathways. Curr. Stem Cell Rep..

[11] Brai E., Marathe S., Astori S., Ben Fredj N., Perry E., Lamy C., Scotti A., Alberi L. (2015). Notch1 Regulates Hippocampal Plasticity Through Interaction with the Reelin Pathway, Glutamatergic Transmission and CREB Signaling. Front. Cell. Neurosci..

[12] Kidd S., Lieber T. (2016). Mechanism of Notch Pathway Activation and Its Role in the Regulation of Olfactory Plasticity in Drosophila melanogaster. PLoS ONE.

[13] Pitulescu M.E., Schmidt I., Giaimo B.D., Antoine T., Berkenfeld F., Ferrante F., Park H., Ehling M., Biljes D., Rocha S.F. (2017). Dll4 and Notch signalling couples sprouting angiogenesis and artery formation. Nat. Cell Biol..

[14] Hasan S.S., Tsaryk R., Lange M., Wisniewski L., Moore J.C., Lawson N.D., Wojciechowska K., Schnittler H., Siekmann A.F. (2017). Endothelial Notch signalling limits angiogenesis via control of artery formation. Nat. Cell Biol..

[15] Aspalter I.M., Gordon E., Dubrac A., Ragab A., Narloch J., Vizán P., Geudens I., Collins R.T., Franco C.A., Abrahams C.L. (2015). Alk1 and Alk5 inhibition by Nrp1 controls vascular sprouting downstream of Notch. Nat. Commun..

[16] Lobov I., Mikhailova N. (2018). The Role of Dll4/Notch Signaling in Normal and Pathological Ocular Angiogenesis: Dll4 Controls Blood Vessel Sprouting and Vessel Remodeling in Normal and Pathological Conditions. J. Ophthalmol..

[17] Antfolk D., Sjöqvist M., Cheng F., Isoniemi K., Duran C.L., Rivero-Muller A., Antila C., Niemi R., Landor S., Bouten C.V.C. (2017). Selective regulation of Notch ligands during angiogenesis is mediated by vimentin. Proc. Natl. Acad. Sci. USA.

[18] Travisano S.I., Oliveira V.L., Prados B., Grego-Bessa J., Piñeiro-Sabarís R., Bou V., Gómez M.J., Sánchez-Cabo F., MacGrogan D., de la Pompa J.L. (2019). Coronary arterial development is regulated by a Dll4-Jag1-EphrinB2 signaling cascade. Elife.

[19] Urbanek K., Lesiak M., Krakowian D., Koryciak-Komarska H., Likus W., Czekaj P., Kusz D., Sieroń A.L. (2017). Notch signaling pathway and gene expression profiles during early in vitro differentiation of liver-derived mesenchymal stromal cells to osteoblasts. Lab. Investig..

[20] Ziouti F., Ebert R., Rummler M., Krug M., Müller-Deubert S., Lüdemann M., Jakob F., Willie B.M., Jundt F. (2019). NOTCH Signaling Is Activated through Mechanical Strain in Human Bone Marrow-Derived Mesenchymal Stromal Cells. Stem Cells Int..

[21] Sjöqvist M., Andersson E.R. (2019). Do as I say, Not(ch) as I do: Lateral control of cell fate. Dev. Biol..

[22] Koon Y.L., Zhang S., Rahmat M.B., Koh C.G., Chiam K.-H. (2018). Enhanced Delta-Notch Lateral Inhibition Model Incorporating Intracellular Notch Heterogeneity and Tension-Dependent Rate of Delta-Notch Binding that Reproduces Sprouting Angiogenesis Patterns. Sci. Rep..

[23] Menchero S., Rollan I., Lopez-Izquierdo A., Andreu M.J., Sainz de Aja J., Kang M., Adan J., Benedito R., Rayon T., Hadjantonakis A.-K. (2019). Transitions in cell potency during early mouse development are driven by Notch. Elife.

[24] Garcıá-Leoń M.J., Fuentes P., de la Pompa J.L., Toribio M.L. (2018). Dynamic regulation of NOTCH1 activation and notch ligand expression in human thymus development. Development.

[25] Mack J.J., Mosqueiro T.S., Archer B.J., Jones W.M., Sunshine H., Faas G.C., Briot A., Aragón R.L., Su T., Romay M.C. (2017). NOTCH1 is a mechanosensor in adult arteries. Nat. Commun..

[26] Luo Z., Shang X., Zhang H., Wang G., Massey P.A., Barton S.R., Kevil C.G., Dong Y. (2019). Notch Signaling in Osteogenesis, Osteoclastogenesis, and Angiogenesis. Am. J. Pathol..

[27] Grazioli P., Felli M.P., Screpanti I., Campese A.F. (2017). The mazy case of Notch and immunoregulatory cells. J. Leukoc. Biol..

[28] Arcaini L., Rossi D., Lucioni M., Nicola M., Bruscaggin A., Fiaccadori V., Riboni R., Ramponi A., Ferretti V.V., Cresta S. (2015). The NOTCH pathway is recurrently mutated in diffuse large B-cell lymphoma associated with hepatitis C virus infection. Haematologica.

[29] Gu Y., Masiero M., Banham A.H. (2016). Notch signaling: Its roles and therapeutic potential in hematological malignancies. Oncotarget.

[30] Takam Kamga P., Collo G.D., Resci F., Bazzoni R., Mercuri A., Quaglia F.M., Tanasi I., Delfino P., Visco C., Bonifacio M. (2019). Notch Signaling Molecules as Prognostic Biomarkers for Acute Myeloid Leukemia. Cancers.

[31] Teodorczyk M., Schmidt M.H.H. (2015). Notching on Cancer’s Door: Notch Signaling in Brain Tumors. Front. Oncol..

[32] Krishna B.M., Jana S., Singhal J., Horne D., Awasthi S., Salgia R., Singhal S.S. (2019). Notch signaling in breast cancer: From pathway analysis to therapy. Cancer Lett..

[33] Xu Y., Wang Y., Liu H., Kang X., Li W., Wei Q. (2016). Genetic variants of genes in the Notch signaling pathway predict overall survival of non-small cell lung cancer patients in the PLCO study. Oncotarget.

[34] Zou B., Zhou X., Lai S., Liu J. (2018). Notch signaling and non-small cell lung cancer (Review). Oncol. Lett..

[35] Chen F., Liu N. (2015). A 10-gene expression signature of Notch pathway predicts recurrence in ovarian carcinoma. Oncol. Lett..

[36] Bhagat T.D., Zou Y., Huang S., Park J., Palmer M.B., Hu C., Li W., Shenoy N., Giricz O., Choudhary G. (2017). Notch Pathway Is Activated via Genetic and Epigenetic Alterations and Is a Therapeutic Target in Clear Cell Renal Cancer. J. Biol. Chem..

[37] Huang Q., Li J., Zheng J., Wei A. (2019). The Carcinogenic Role of the Notch Signaling Pathway in the Development of Hepatocellular Carcinoma. J. Cancer.

[38] Zhang Y., Li D., Feng F., An L., Hui F., Dang D., Zhao Q. (2017). Progressive and Prognosis Value of Notch Receptors and Ligands in Hepatocellular Carcinoma: A Systematic Review and Meta-analysis. Sci. Rep..

[39] Kałafut J., Czapiński J., Przybyszewska-Podstawka A., Czerwonka A., Odrzywolski A., Sahlgren C., Rivero-Müller A. (2022). Optogenetic control of NOTCH1 signaling. Cell Commun. Signal..

[40] Chen Y.M., Liu Y., Wei H.Y., Lv K.Z., Fu P.F. (2016). Large intergenic non-coding RNA-ROR reverses Gemcitabineinduced autophagy and apoptosis in breast cancer cells. Oncotarget.

[41] Li D., Xu D., Zhang Y., Chen P., Xie J. (2022). Effect of Notch1 signaling on cellular proliferation and apoptosis in human laryngeal carcinoma. World J. Surg. Oncol..

[42] Li Z.-L., Chen C., Yang Y., Wang C., Yang T., Yang X., Liu S.-C. (2015). Gamma secretase inhibitor enhances sensitivity to doxorubicin in MDA-MB-231 cells. Int. J. Clin. Exp. Pathol..

[43] Zang S., Chen F., Dai J., Guo D., Tse W., Qu X., Ma D., Ji C. (2010). RNAi-mediated knockdown of Notch-1 leads to cell growth inhibition and enhanced chemosensitivity in human breast cancer. Oncol. Rep..

[44] Mao J., Song B., Shi Y., Wang B., Fan S., Yu X., Tang J., Li L. (2013). ShRNA targeting Notch1 sensitizes breast cancer stem cell to paclitaxel. Int. J. Biochem. Cell Biol..

[45] Lee S.H., Do S.I., Lee H.J., Kang H.J., Koo B.S., Lim Y.C. (2016). Notch1 signaling contributes to stemness in head and neck squamous cell carcinoma. Lab. Investig..

[46] Katarkar A., Bottoni G., Clocchiatti A., Goruppi S., Bordignon P., Lazzaroni F., Gregnanin I., Ostano P., Neel V., Dotto G.P. (2020). NOTCH1 gene amplification promotes expansion of Cancer Associated Fibroblast populations in human skin. Nat. Commun..

[47] Misiorek J.O., Przybyszewska-Podstawka A., Kałafut J., Paziewska B., Rolle K., Rivero-Müller A., Nees M. (2021). Context Matters: NOTCH Signatures and Pathway in Cancer Progression and Metastasis. Cells.

[48] Aster J.C., Pear W.S., Blacklow S.C. (2017). The Varied Roles of Notch in Cancer. Annu. Rev. Pathol..

[49] Meisel C.T., Porcheri C., Mitsiadis T.A. (2020). Cancer Stem Cells, Quo Vadis? The Notch Signaling Pathway in Tumor Initiation and Progression. Cells.

[50] Lee B., Lee S., Shim J. (2021). YTHDF2 Suppresses Notch Signaling through Post-transcriptional Regulation on Notch1. Int. J. Biol. Sci..

[51] Salviano-Silva A., Berti F.C.B., Lobo-Alves S.C., de Araujo-Souza P.S., Boldt A.B.W., Malheiros D. (2020). Interaction of Long Noncoding RNAs and Notch Signaling: Implications for Tissue Homeostasis Loss. Notch Signaling in Embryology and Cancer.

[52] Shen Y., Li C., Zhou L., Huang J. (2020). G protein-coupled oestrogen receptor promotes cell growth of non-small cell lung cancer cells via YAP1/QKI/circNOTCH1/m6A methylated NOTCH1 signalling. J. Cell. Mol. Med..

[53] Wu X., Ye W., Gong Y. (2022). The Role of RNA Methyltransferase METTL3 in Normal and Malignant Hematopoiesis. Front. Oncol..

[54] Antfolk D., Antila C., Kemppainen K., Landor S.K.J., Sahlgren C. (2019). Decoding the PTM-switchboard of Notch. Biochim. Biophys. Acta Mol. Cell Res..

[55] Li N., Fassl A., Chick J., Inuzuka H., Li X., Mansour M.R., Liu L., Wang H., King B., Shaik S. (2014). Cyclin C is a haploinsufficient tumour suppressor. Nat. Cell Biol..

[56] Ranganathan P., Vasquez-Del Carpio R., Kaplan F.M., Wang H., Gupta A., VanWye J.D., Capobianco A.J. (2011). Hierarchical phosphorylation within the ankyrin repeat domain defines a phosphoregulatory loop that regulates notch transcriptional activity. J. Biol. Chem..

[57] Santio N.M., Landor S.K.J., Vahtera L., Ylä-Pelto J., Paloniemi E., Imanishi S.Y., Corthals G., Varjosalo M., Manoharan G.B., Uri A. (2016). Phosphorylation of Notch1 by Pim kinases promotes oncogenic signaling in breast and prostate cancer cells. Oncotarget.

[58] Song J., Park S., Kim M., Shin I. (2008). Down-regulation of Notch-dependent transcription by Akt in vitro. FEBS Lett..

[59] Pettersson S., Sczaniecka M., McLaren L., Russell F., Gladstone K., Hupp T., Wallace M. (2013). Non-degradative ubiquitination of the Notch1 receptor by the E3 ligase MDM2 activates the Notch signalling pathway. Biochem. J..

[60] Zhang P., Wu W., Chen Q., Chen M. (2019). Non-Coding RNAs and their Integrated Networks. J. Integr. Bioinform..

[61] Du Y., Chen H., Li X.-F., Lu Y.-C., Feng Y.-B., Zhang L. (2019). Molecular mechanism of Notch signaling with special emphasis on microRNAs: Implications for glioma. J. Cell. Physiol..

[62] Majidinia M., Darband S.G., Kaviani M., Nabavi S.M., Jahanban-Esfahlan R., Yousefi B. (2018). Cross-regulation between Notch signaling pathway and miRNA machinery in cancer. DNA Repair.

[63] Ghafouri-Fard S., Glassy M.C., Abak A., Hussen B.M., Niazi V., Taheri M. (2021). The interaction between miRNAs/lncRNAs and Notch pathway in human disorders. Biomed. Pharmacother..

[64] Chen C.-H., Pan C.-Y., Lin W. (2019). Overlapping protein-coding genes in human genome and their coincidental expression in tissues. Sci. Rep..

[65] Laurent G.S., Wahlestedt C., Kapranov P. (2016). The Landscape of long non-coding RNA classification Georges. Trends Genet..

[66] Statello L., Guo C.J., Chen L.L., Huarte M. (2021). Gene regulation by long non-coding RNAs and its biological functions. Nat. Rev. Mol. Cell Biol..

[67] Fernandes J.C.R., Acuña S.M., Aoki J.I., Floeter-Winter L.M., Muxel S.M. (2019). Long non-coding RNAs in the regulation of gene expression: Physiology and disease. Non Coding RNA.

[68] Karakas D., Ozpolat B. (2021). The Role of LncRNAs in Translation. Non Coding RNA.

[69] Pisignano G., Pavlaki I., Murrell A. (2019). Being in a loop: How long non-coding RNAs organise genome architecture. Essays Biochem..

[70] Mishra K., Kanduri C. (2019). Understanding Long Noncoding RNA and Chromatin Interactions: What We Know So Far. Non Coding RNA.

[71] Peng W.-X., Koirala P., Mo Y.-Y. (2017). LncRNA-mediated regulation of cell signaling in cancer. Oncogene.

[72] Khorkova O., Hsiao J., Wahlestedt C. (2015). Basic biology and therapeutic implications of lncRNA. Adv. Drug Deliv. Rev..

[73] Marchese F.P., Raimondi I., Huarte M. (2017). The multidimensional mechanisms of long noncoding RNA function. Genome Biol..

[74] Gazave E., Lapébie P., Richards G.S., Brunet F., Ereskovsky A.V., Degnan B.M., Borchiellini C., Vervoort M., Renard E. (2009). Origin and evolution of the Notch signalling pathway: An overview from eukaryotic genomes. BMC Evol. Biol..

[75] Salazar J.L., Yamamoto S. (2018). Integration of Drosophila and Human Genetics to Understand Notch Signaling Related Diseases.

[76] Meurette O., Mehlen P. (2018). Notch Signaling in the Tumor Microenvironment. Cancer Cell.

[77] Lambertini C., Pantano S., Dotto G.P. (2010). Differential Control of Notch1 Gene Transcription by Klf4 and Sp3 Transcription Factors in Normal versus Cancer-Derived Keratinocytes. PLoS ONE.

[78] Wang Y., Wu P., Lin R., Rong L., Xue Y., Fang Y. (2015). LncRNA NALT interaction with NOTCH1 promoted cell proliferation in pediatric T cell acute lymphoblastic leukemia. Sci. Rep..

[79] Piao H.Y., Guo S., Wang Y., Zhang J. (2019). Long noncoding RNA NALT1-induced gastric cancer invasion and metastasis via NOTCH signaling pathway. World J. Gastroenterol..

[80] Kwon H., Song K., Han C., Zhang J., Lu L., Chen W., Wu T. (2017). Epigenetic Silencing of miRNA-34a in Human Cholangiocarcinoma via EZH2 and DNA Methylation: Impact on Regulation of Notch Pathway. Am. J. Pathol..

[81] Li Y., Tzatzalos E., Kwan K.Y., Grumet M., Cai L. (2016). Transcriptional Regulation of Notch1 Expression by Nkx6.1 in Neural Stem/Progenitor Cells during Ventral Spinal Cord Development. Sci. Rep..

[82] Zhang F.F., Liu Y.H., Wang D.W., Liu T.S., Yang Y., Guo J.M., Pan Y., Zhang Y.F., Du H., Li L. (2020). Obesity-induced reduced expression of the lncRNA ROIT impairs insulin transcription by downregulation of Nkx6.1 methylation. Diabetologia.

[83] Huang S., Li C., Huang J., Luo P., Mo D., Wang H. (2020). LncRNA FEZF1-AS1 promotes non-small lung cancer cell migration and invasion through the up-regulation of NOTCH1 by serving as a sponge of miR-34a. BMC Pulm. Med..

[84] Sun X., Huang T., Liu Z., Sun M., Luo S. (2019). LncRNA SNHG7 contributes to tumorigenesis and progression in breast cancer by interacting with miR-34a through EMT initiation and the Notch-1 pathway. Eur. J. Pharmacol..

[85] Luo Y.Y., Wang Z.H., Yu Q., Yuan L.L., Peng H.L., Xu Y.X. (2020). LncRNA-NEAT1 promotes proliferation of T-ALL cells via miR-146b-5p/NOTCH1 signaling pathway. Pathol. Res. Pract..

[86] Rani N., Nowakowski T.J., Zhou H., Godshalk S.E., Lisi V., Kriegstein A.R., Kosik K.S. (2016). A Primate lncRNA Mediates Notch Signaling during Neuronal Development by Sequestering miRNA. Neuron.

[87] Zhang X., Wu J., Wu C., Chen W., Lin R., Zhou Y., Huang X. (2018). The LINC01138 interacts with PRMT5 to promote SREBP1-mediated lipid desaturation and cell growth in clear cell renal cell carcinoma. Biochem. Biophys. Res. Commun..

[88] Xu T.P., Ma P., Wang W.Y., Shuai Y., Wang Y.F., Yu T., Xia R., Shu Y.Q. (2019). KLF5 and MYC modulated LINC00346 contributes to gastric cancer progression through acting as a competing endogeous RNA and indicates poor outcome. Cell Death Differ..

[89] Xu Z., Huang B., Zhang Q., He X., Wei H., Zhang D. (2019). NOTCH1 regulates the proliferation and migration of bladder cancer cells by cooperating with long non-coding RNA HCG18 and microRNA-34c-5p. J. Cell. Biochem..

[90] Ke J., Shen Z., Hu W., Li M., Shi Y., Xie Z., Wu D. (2020). LncRNA DCST1-AS1 was upregulated in endometrial carcinoma and may sponge miR-92a-3p to upregulate notch1. Cancer Manag. Res..

[91] Ounzain S., Micheletti R., Arnan C., Plaisance I., Cecchi D., Schroen B., Reverter F., Alexanian M., Gonzales C., Ng S.Y. (2015). CARMEN, a human super enhancer-associated long noncoding RNA controlling cardiac specification, differentiation and homeostasis. J. Mol. Cell. Cardiol..

[92] Plaisance I., Perruchoud S., Fernandez-Tenorio M., Gonzales C., Ounzain S., Ruchat P., Nemir M., Niggli E., Pedrazzini T. (2016). Cardiomyocyte Lineage Specification in Adult Human Cardiac Precursor Cells Via Modulation of Enhancer-Associated Long Noncoding RNA Expression. JACC Basic Transl. Sci..

[93] Kolenda T., Guglas K., Kopczyńska M., Teresiak A., Bliźniak R., Mackiewicz A., Lamperska K., Mackiewicz J. (2019). Oncogenic Role of ZFAS1 lncRNA in Head and Neck Squamous Cell Carcinomas. Cells.

[94] Deng X., Lin Z., Zuo C., Fu Y. (2020). Upregulation of miR-150-5p alleviates LPS-induced inflammatory response and apoptosis of RAW264.7 macrophages by targeting Notch1. Open Life Sci..

[95] Zhao J., Liu H.R. (2019). Down-regulation of long noncoding RNA DLX6-AS1 defines good prognosis and inhibits proliferation and metastasis in human epithelial ovarian cancer cells via Notch signaling pathway. Eur. Rev. Med. Pharmacol. Sci..

[96] An Y., Chen X.M., Yang Y., Mo F., Jiang Y., Sun D.L., Cai H.H. (2018). LncRNA DLX6-AS1 promoted cancer cell proliferation and invasion by attenuating the endogenous function of miR-181b in pancreatic cancer 11 Medical and Health Sciences 1112 Oncology and Carcinogenesis. Cancer Cell Int..

[97] Ni R., Huang Y., Wang J., Liu Y. (2019). Knockdown of lncRNA DLX6-AS1 inhibits cell proliferation, migration and invasion while promotes apoptosis by downregulating PRR11 expression and upregulating miR-144 in non-small cell lung cancer. Biomed. Pharmacother..

[98] Li X., Zhang H., Wu X. (2019). Long noncoding RNA DLX6-AS1 accelerates the glioma carcinogenesis by competing endogenous sponging miR-197-5p to relieve E2F1. Gene.

[99] Petrovic J., Zhou Y., Fasolino M., Goldman N., Schwartz G.W., Mumbach M.R., Nguyen S.C., Rome K.S., Sela Y., Zapataro Z. (2019). Oncogenic Notch Promotes Long-Range Regulatory Interactions within Hyperconnected 3D Cliques. Mol. Cell.

[100] Liu X., Xiao Z.D., Han L., Zhang J., Lee S.W., Wang W., Lee H., Zhuang L., Chen J., Lin H.K. (2016). LncRNA NBR2 engages a metabolic checkpoint by regulating AMPK under energy stress. Nat. Cell Biol..

[101] Gao Y.P., Li Y., Li H.J., Zhao B. (2019). LncRNA NBR2 inhibits EMT progression by regulating Notch 1 pathway in NSCLC. Eur. Rev. Med. Pharmacol. Sci..

[102] Ellisen L.W., Bird J., West D.C., Soreng A.L., Reynolds T.C., Smith S.D., Sklar J. (1991). TAN-1, the human homolog of the Drosophila notch gene, is broken by chromosomal translocations in T lymphoblastic neoplasms. Cell.

[103] Trimarchi T., Bilal E., Ntziachristos P., Fabbri G., Dalla-Favera R., Tsirigos A., Aifantis I. (2014). Genome-wide mapping and characterization of notch-regulated long noncoding RNAs in acute leukemia. Cell.

[104] Qian J., Garg A., Li F., Shen Q., Xiao K. (2020). lncRNA LUNAR1 accelerates colorectal cancer progression by targeting the miR 495 3p/MYCBP axis. Int. J. Oncol..

[105] Chen Q., Chen X.F., Li D.E., Yang L., Xu L.P., Hu Y., Jiang J.S. (2019). Long non-coding RNA MACC1-AS1 promoted pancreatic carcinoma progression through activation of PAX8/NOTCH1 signaling pathway. J. Exp. Clin. Cancer Res..

[106] Cai Z., Zhao B., Deng Y., Shangguan S., Zhou F., Zhou W., Li X., Li Y., Chen G. (2016). Notch signaling in cerebrovascular diseases (Review). Mol. Med. Rep..

[107] Malashicheva A., Kostina A., Kostareva A., Irtyuga O., Uspensky V. (2019). Notch signaling in the pathogenesis of thoracic aortic aneurysms: A bridge between embryonic and adult states. Biochim. Biophys. Acta Mol. Basis Dis..

[108] MacGrogan D., Münch J., de la Pompa J.L. (2018). Notch and interacting signalling pathways in cardiac development, disease, and regeneration. Nat. Rev. Cardiol..

[109] Ho D.M., Artavanis-Tsakonas S., Louvi A. (2020). The Notch pathway in CNS homeostasis and neurodegeneration. WIREs Dev. Biol..

[110] Smith K.A., Voiriot G., Tang H., Fraidenburg D.R., Song S., Yamamura H., Yamamura A., Guo Q., Wan J., Pohl N.M. (2015). Notch Activation of Ca^2+^ Signaling in the Development of Hypoxic Pulmonary Vasoconstriction and Pulmonary Hypertension. Am. J. Respir. Cell Mol. Biol..

[111] Turnpenny P.D., Alman B., Cornier A.S., Giampietro P.F., Offiah A., Tassy O., Pourquié O., Kusumi K., Dunwoodie S. (2007). Abnormal vertebral segmentation and the notch signaling pathway in man. Dev. Dyn. Off. Publ. Am. Assoc. Anat..

[112] Servián-Morilla E., Takeuchi H., Lee T.V., Clarimon J., Mavillard F., Area-Gómez E., Rivas E., Nieto-González J.L., Rivero M.C., Cabrera-Serrano M. (2016). A POGLUT 1 mutation causes a muscular dystrophy with reduced Notch signaling and satellite cell loss. EMBO Mol. Med..

[113] Karaca E., Yuregir O.O., Bozdogan S.T., Aslan H., Pehlivan D., Jhangiani S.N., Akdemir Z.C., Gambin T., Bayram Y., Atik M.M. (2015). Rare variants in the notch signaling pathway describe a novel type of autosomal recessive Klippel-Feil syndrome. Am. J. Med. Genet. Part A.

[114] Adami G., Rossini M., Gatti D., Orsolini G., Idolazzi L., Viapiana O., Scarpa A., Canalis E. (2016). Hajdu Cheney Syndrome; report of a novel NOTCH2 mutation and treatment with denosumab. Bone.

[115] Mašek J., Andersson E.R. (2017). The developmental biology of genetic Notch disorders. Development.

[116] Arreola A., Payne L.B., Julian M.H., de Cubas A.A., Daniels A.B., Taylor S., Zhao H., Darden J., Bautch V.L., Rathmell W.K. (2018). Von Hippel-Lindau mutations disrupt vascular patterning and maturation via Notch. JCI Insight.

[117] Mitchell E., Gilbert M., Loomes K.M. (2018). Alagille Syndrome. Clin. Liver Dis..

[118] Wasson C.W., Abignano G., Hermes H., Malaab M., Ross R.L., Jimenez S.A., Chang H.Y., Feghali-Bostwick C.A., Del Galdo F. (2020). Long non-coding RNA HOTAIR drives EZH2-dependent myofibroblast activation in systemic sclerosis through miRNA 34a-dependent activation of NOTCH. Ann. Rheum. Dis..

[119] Li H., Li D., Meng N. (2017). Effects of RUNX3 mediated Notch signaling pathway on biological characteristics of colorectal cancer cells. Int. J. Oncol..

[120] Wang Y., Yang X., Jiang A., Wang W., Li J., Wen J. (2019). Methylation-dependent transcriptional repression of RUNX3 by KCNQ1OT1 regulates mouse cardiac microvascular endothelial cell viability and inflammatory response following myocardial infarction. FASEB J..

[121] Wasson C.W., Ross R.L., Wells R., Corinaldesi C., Georgiou I.C., Riobo-Del Galdo N.A., Del Galdo F. (2020). Long non-coding RNA HOTAIR induces GLI2 expression through Notch signalling in systemic sclerosis dermal fibroblasts. Arthritis Res. Ther..

[122] Liu Y., Sun M., Xia R., Zhang E., Liu X., Zhang Z., Xu T., De W., Liu B., Wang Z. (2015). LincHOTAIR epigenetically silences miR34a by binding to PRC2 to promote the epithelial-to-mesenchymal transition in human gastric cancer. Cell Death Dis..

[123] Zhang M., Liu H.Y., Han Y.L., Wang L., Zhai D.D., Ma T., Zhang M.J., Liang C.Z., Shen Y. (2019). Silence of lncRNA XIST represses myocardial cell apoptosis in rats with acute myocardial infarction through regulating MIR-449. Eur. Rev. Med. Pharmacol. Sci..

[124] Hadji F., Boulanger M.C., Guay S.P., Gaudreault N., Amellah S., Mkannez G., Bouchareb R., Marchand J.T., Nsaibia M.J., Guauque-Olarte S. (2016). Altered DNA Methylation of Long Noncoding RNA H19 in Calcific Aortic Valve Disease Promotes Mineralization by Silencing NOTCH1. Circulation.

[125] Wang J., Cao B., Zhao H., Gao Y., Luo Y., Chen Y., Feng J. (2019). Long noncoding RNA H19 prevents neurogenesis in ischemic stroke through p53/Notch1 pathway. Brain Res. Bull..

[126] Wan Y., Yang Z.Q. (2020). LncRNA NEAT1 affects inflammatory response by targeting miR-129-5p and regulating Notch signaling pathway in epilepsy. Cell Cycle.

[127] Wei R., Chen Y., Zhao Z., Gu Q., Wu J. (2019). LncRNA FAM83H-AS1 induces nucleus pulposus cell growth via targeting the Notch signaling pathway. J. Cell. Physiol..

[128] Liu Z.B., Tang C., Jin X., Liu S.H., Pi W. (2018). Increased expression of lncRNA SNHG12 predicts a poor prognosis of nasopharyngeal carcinoma and regulates cell proliferation and metastasis by modulating Notch signal pathway. Cancer Biomark..

[129] Chen M., Xia Z., Chen C., Hu W., Yuan Y. (2018). LncRNA MALAT1 promotes epithelial-to-mesenchymal transition of esophageal cancer through Ezh2-Notch1 signaling pathway. Anti Cancer Drugs.

[130] Zhang Y., Jin X., Wang Z., Zhang X., Liu S., Liu G. (2017). Downregulation of SNHG1 suppresses cell proliferation and invasion by regulating Notch signaling pathway in esophageal squamous cell cancer. Cancer Biomark..

[131] Liu T., Zuo J.J., Li F., Xu Y.C., Zheng A.Y., Tao Z.Z. (2019). LncRNA SNHG1 promotes cell proliferation in laryngeal cancer via Notch1 signaling pathway. Eur. Rev. Med. Pharmacol. Sci..

[132] Lu S., Dong W., Zhao P., Liu Z. (2018). LncRNA FAM83H-AS1 is associated with the prognosis of colorectal carcinoma and promotes cell proliferation by targeting the notch signaling pathway. Oncol. Lett..

[133] Tang D., Yang Z., Long F., Luo L., Yang B., Zhu R., Sang X., Cao G., Wang K. (2019). Long noncoding RNA MALAT1 mediates stem cell-like properties in human colorectal cancer cells by regulating miR-20b-5p/Oct4 axis. J. Cell. Physiol..

[134] Zhang H.F., Li W., Han Y.D. (2018). LINC00261 suppresses cell proliferation, invasion and Notch signaling pathway in hepatocellular carcinoma. Cancer Biomark..

[135] Bai L., Wang A., Zhang Y., Xu X., Zhang X. (2018). Knockdown of MALAT1 enhances chemosensitivity of ovarian cancer cells to cisplatin through inhibiting the Notch1 signaling pathway. Exp. Cell Res..

[136] Guo Q., Qian Z., Yan D., Li L., Huang L. (2016). LncRNA-MEG3 inhibits cell proliferation of endometrial carcinoma by repressing Notch signaling. Biomed. Pharmacother..

[137] Wu Z., Huang W., Chen Y., Chen B., Liu R., Bai P., Xing J. (2019). LINC01638 lncRNA promotes the proliferation, migration and invasion of prostate carcinoma cells by interacting with Notch1. Cancer Biomark..

[138] Zhu Y., Tong Y., Wu J., Liu Y., Zhao M. (2019). Knockdown of LncRNA GHET1 suppresses prostate cancer cell proliferation by inhibiting HIF-1α/Notch-1 signaling pathway via KLF2. BioFactors.

[139] Tang L.X., Su S.F., Wan Q., He P., Xhang Y., Cheng X.M. (2019). Novel long non-coding RNA LBX2-AS1 indicates poor prognosis and promotes cell proliferation and metastasis through Notch signaling in non-small cell lung cancer. Eur. Rev. Med. Pharmacol. Sci..

[140] Qi H., Wang S., Wu J., Yang S., Gray S., Ng C.S.H., Du J., Underwood M.J., Li M.Y., Chen G.G. (2019). EGFR-AS1/HIF2A regulates the expression of FOXP3 to impact the cancer stemness of smoking-related non-small cell lung cancer. Ther. Adv. Med. Oncol..

[141] Li S., Zhao H., Li J., Zhang A., Wang H. (2018). Downregulation of long non-coding RNA LET predicts poor prognosis and increases Notch signaling in non-small cell lung cancer. Oncotarget.

[142] Zhang S.Z., Cai L., Li B. (2017). MEG3 long non-coding RNA prevents cell growth and metastasis of osteosarcoma. Bratisl. Med. J..

[143] Cai W., Wu B., Li Z., He P., Wang B., Cai A., Zhang X. (2019). LncRNA NBR2 inhibits epithelial-mesenchymal transition by regulating Notch1 signaling in osteosarcoma cells. J. Cell. Biochem..

[144] Li Z., Tang Y., Xing W., Dong W., Wang Z. (2018). LncRNA, CRNDE promotes osteosarcoma cell proliferation, invasion and migration by regulating Notch1 signaling and epithelial-mesenchymal transition. Exp. Mol. Pathol..

[145] Vander Roest M., Krapp C., Thorvaldsen J.L., Bartolomei M.S., Merryman W.D. (2019). H19 is not hypomethylated or upregulated with age or sex in the aortic valves of mice. Physiol. Rep..

[146] Xu S., Chen L., Tang Y., Yuan P., Yan J., Zheng Y., Huang L., Li Z., Sun Y., Han S. (2019). Lnc-RP5 Regulates the miR-129-5p/Notch1/PFV Internal Promoter Axis to Promote the Expression of the Prototype Foamy Virus Transactivator Tas. Virol. Sin..

[147] Zhang K., Han X., Zhang Z., Zheng L., Hu Z., Yao Q., Cui H., Shu G., Si M., Li C. (2017). The liver-enriched lnc-LFAR1 promotes liver fibrosis by activating TGFβ and Notch pathways. Nat. Commun..

[148] Du Z., Fei T., Verhaak R.G.W., Su Z., Zhang Y., Brown M., Chen Y., Liu X.S. (2013). Integrative genomic analyses reveal clinically relevant long noncoding RNAs in human cancer. Nat. Struct. Mol. Biol..

[149] Pardini B., Sabo A.A., Birolo G., Calin G.A. (2019). Noncoding rnas in extracellular fluids as cancer biomarkers: The new frontier of liquid biopsies. Cancers.

[150] Zeuschner P., Linxweiler J., Junker K. (2020). Non-coding RNAs as biomarkers in liquid biopsies with a special emphasis on extracellular vesicles in urological malignancies. Expert Rev. Mol. Diagn..

[151] Bolha L., Ravnik-Glavač M., Glavač D. (2017). Long Noncoding RNAs as Biomarkers in Cancer. Dis. Markers.

[152] Chandra Gupta S., Nandan Tripathi Y. (2017). Potential of long non-coding RNAs in cancer patients: From biomarkers to therapeutic targets. Int. J. Cancer.

[153] Deng J., Tang J., Wang G., Zhu Y.S. (2017). Long non-coding RNA as potential biomarker for prostate cancer: Is it making a difference?. Int. J. Environ. Res. Public Health.

[154] McCleland M.L., Mesh K., Lorenzana E., Chopra V.S., Segal E., Watanabe C., Haley B., Mayba O., Yaylaoglu M., Gnad F. (2016). CCAT1 is an enhancer-templated RNA that predicts BET sensitivity in colorectal cancer. J. Clin. Investig..

[155] Barrows J.K., Lin B., Quaas C.E., Fullbright G., Wallace E.N., Long D.T. (2022). BRD4 promotes resection and homology-directed repair of DNA double-strand breaks. Nat. Commun..

[156] Donati B., Lorenzini E., Ciarrocchi A. (2018). BRD4 and Cancer: Going beyond transcriptional regulation. Mol. Cancer.

[157] Kulikowski E., Rakai B.D., Wong N.C.W. (2021). Inhibitors of bromodomain and extra-terminal proteins for treating multiple human diseases. Med. Res. Rev..

[158] Hang Q., Sun R., Jiang C., Li Y. (2015). Notch 1 promotes cisplatin-resistant gastric cancer formation by upregulating lncRNA AK022798 expression. Anticancer Drugs.

[159] Yan L., Zheng M., Wang H. (2019). Circular RNA hsa_circ_0072309 inhibits proliferation and invasion of breast cancer cells via targeting miR-492. Cancer Manag. Res..

[160] Smid M., Wilting S.M., Uhr K., Rodríguez-González F.G., De Weerd V., Prager-Van Der Smissen W.J.C., Van Der Vlugt-Daane M., Van Galen A., Nik-Zainal S., Butler A. (2019). The circular RNome of primary breast cancer. Genome Res..

[161] Xu H., Zhang Y., Qi L., Ding L., Jiang H., Yu H. (2018). NFIX Circular RNA Promotes Glioma Progression by Regulating miR-34a-5p via Notch Signaling Pathway. Front. Mol. Neurosci..

[162] Chen Y., Li Z., Zhang M., Wang B., Ye J., Zhang Y., Tang D., Ma D., Jin W., Li X. (2019). Circ-ASH2L promotes tumor progression by sponging miR-34a to regulate Notch1 in pancreatic ductal adenocarcinoma. J. Exp. Clin. Cancer Res..

[163] Allen F., Maillard I. (2021). Therapeutic Targeting of Notch Signaling: From Cancer to Inflammatory Disorders. Front. Cell Dev. Biol..

