# Supplementary material for: Regulation of Notch1 Signalling by Long Non-Coding RNAs in Cancers and Other Health Disorders
Source: Int J Mol Sci. 2023 Aug 8;24(16):12579. doi: 10.3390/ijms241612579 (PMC10454443; doi:10.3390/ijms241612579)
Supplement: Supplementary file 1 [file ijms-24-12579-s001.zip › ijms-2529127-supplementary.pdf]

Table S1. Interaction between lncRNAs and Notch1 signalling in various health disorders.

Localisation	Disease	lncRNA	NOTCH1 expression	Cell line	Animal models	Patients	Reference
Cardiac system	Acute myocardial infarction	*XIST*↑	↑	-	AMI rat model	-	[1]
	Calcific aortic valve disease	*H19*↑	↓	VICs, Saos2, COS7	-	36 patients	[2]
	Myocardial infarction	*KCNQ1 OT1*↑	↑	-	C57BL/ 6 male mice	-	[3]
	Ischemic stroke	*H19*↑	↓	-	male C57BL/ 6 J mice	40 patients	[4]
Immune system	T-ALL	*NALT1*↑	↑	Jurkat cells	-	Bone marrow of 20 children	[5]
	Systemic sclerosis (SSc)	*HOTAI R*↑	↑	-	-	12 adult patients	[6]
Neural system	Epilepsy	*NEAT1*↑	↑	CTX-TNA	-	6 patients	[7]
	Intervertebral disc degeneration	*FAM83 H-AS1*↑	↑	-	-	10 patients	[8]
	Nasopharynge al carcinoma	*SNHG1 2*↑	↑	SUNE1, CNE1, CNE2 68, HNE-1	-	139 tissue samples	[9]
Head and neck cancer	Esophageal cancer	*MALAT 1*↑	↑	TE-1, EC109, KYSE30, OE21	-	-	[10]
		SNHG1 ↑	↑	Eca109, TE-1	-	72 patients	[11]
	Laryngeal cancer	*SNHG1* ↑	↑	-	-	42 patients (different tumour stages)	[12]
Digestive system cancer	Pancreatic carcinoma	*MACC1 -AS1*↑	↑	BxPC-3, PANC-1, MIA PaCa-2, KP-2, AsPC-1, Capan-1	-	2 cohorts (98 and 124 patients) primary tissues	[13]

	Gastric cancer	*NALT1* (*LINC01573*) ↑	↑	SGC-7901, BGC-823	-	336 patients after D2 lymph node dissected gastrectomy	[14]
		LINC00346↑ (sponge for *miR-34a-5p*)	↑	MGC803, BGC823, MKN28, MKN45, SGC7901	Xenografts in athymic (nu/nu) mouse	58 gastric adenocarcinoma tissue samples	[15]
	Colorectal carcinoma	*FAM83H-AS1*↑	↑	SW480, LoVo, HCT116, HT29	-	40 patients	[16]
		MALAT1↑	↑	COLO205, HCT-116, LoVo, HT26, SW480	nude Balb/c mice	-	[17]
	Hepatocellular carcinoma	*LINC00261*↓	↑	SMCC-7721, MHCC97L, MHCC97H	-	66 tissue samples	[18]
Reproductive tract	Ovarian cancer	*DLX6-AS1*↑	↑	HEY, SKOV3, OVCAR-3	-	128 tissue samples	[19]
		MALAT1↑	↑	A2780, OVCAR3, COC1, A2780/CDDP, COC1/CDDP, OVCAR3/DDP	-	20 paired tumour tissue samples	[20]
	Endometrial carcinoma (EC)	*MEG3*↓	↑	HEC-1A, KLE	-	30 tumour tissue samples	[21]
		DCST1-AS1↑	↑	HEC-1	-	62 patients	[22]
	Prostate carcinoma	*LINCO1638*↑	↑	-	-	42 patients	[23]

		GHET1 ↑	↑	LNCap, C4-2	-	30 patients	[24]
Other cancers	Breast cancer	*linc-ROR*↑	↓	MDA-MB-231	-	-	[25]
		SNHG7 ↑	↑	-	-	37 pairs of tumour tissue samples	[26]
	Lung cancer	*NBR2*↓	↑	-	-	50 NSCLC patients tissue samples	[27]
		LBX2-AS1↑	↑	A549, PC9, H1975, SPC-A1, H1299	-	165 NSCLC patients	[28]
		EGFR-AS1↓	↑	NCH-H460, NCH-H23	-	87 NSCLC patients	[29]
		LET↓	↑	A549, 95D, NCI-H292, NCI-H1975	-	66 NSCLC patients	[30]
	Osteosarcoma	*MEG3*↓	↑	MG-63, U2OS	-	-	[31]
		NBR2↓	↑	MG-63, U2OS, SAOS-2	-	62 patients	[32]
		CRNDE ↑	↑	MG-63, SAOS-2, U2OS	-	72 patients	[33]

Abbreviations: ↑ represents upregulation, ↓ represents downregualation.
